# From Powders to Dense Metal Parts: Characterization of a Commercial AlSiMg Alloy Processed through Direct Metal Laser Sintering

**DOI:** 10.3390/ma6030856

**Published:** 2013-03-06

**Authors:** Diego Manfredi, Flaviana Calignano, Manickavasagam Krishnan, Riccardo Canali, Elisa Paola Ambrosio, Eleonora Atzeni

**Affiliations:** 1 Center for Space Human Robotics @Polito, Istituto Italiano di Tecnologia, Corso Trento 21, Torino 10129, Italy; E-Mails: flaviana.calignano@iit.it (F.C.); manickavasagam.krishnan@iit.it (M.K.); riccardo.canali@iit.it (R.C.); elisa.ambrosio@iit.it (E.P.A.); 2 Department of Management and Production Engineering, Politecnico di Torino, Corso Duca degli Abruzzi 24, Torino 10129, Italy; E-Mail: eleonora.atzeni@polito.it; 3 Department of Applied Science and Technology, Politecnico di Torino, Corso Duca degli Abruzzi 24, Torino 10129, Italy

**Keywords:** additive manufacturing (AM), direct metal laser sintering (DMLS), aluminum alloys, light microscopy, electron microscopy, mechanical characterization

## Abstract

In this paper, a characterization of an AlSiMg alloy processed by direct metal laser sintering (DMLS) is presented, from the analysis of the starting powders, in terms of size, morphology and chemical composition, through to the evaluation of mechanical and microstructural properties of specimens built along different orientations parallel and perpendicular to the powder deposition plane. With respect to a similar aluminum alloy as-fabricated, a higher yield strength of about 40% due to the very fine microstructure, closely related to the mechanisms involved in this additive process is observed.

## 1. Introduction

Additive manufacturing (AM) of metal end-usable parts is well recognized as an interesting alternative to other conventional or unconventional processes for medium batch production, thanks to its capability to produce complex shapes and integrated parts of a high strength-to-weight ratio [1]. Furthermore, AM techniques have the potential to achieve zero wastage through the use of recycling within the processes. This also results in a reduction in emissions, because fewer raw materials need to be produced. Usually, in molding processes, e.g., die casting, lots of energy and resources are consumed to produce tools like dies and moulds. By contrast AM techniques provide almost unchallenged freedom for design without the need for part-specific tooling [2]. Moreover, in comparison to conventional manufacturing technologies AM techniques do not directly use toxic chemicals, such as lubricant or coolant [3]. Additive technologies directly translate virtual three-dimensional models into physical parts in a quick and easy process. Basically the data is sliced into a series of thin sections, then combined into the AM machine, which subsequently adds them together in a layered sequence [4]. As reported in literature, available AM techniques for the production of metal parts use an energy beam source to create the sections by locally and selectively melting a powder bed [5]. Different approaches can be distinguished:

• Indirect processing, using metal powders mixed with polymer binders;
• Liquid-phase sintering, using a mixture of two metal powders or a metal alloy;
• Full melting, the most recently developed method, using a single metal powder that is fully melted, as the name implies.

The result of the first two approaches is a two-phase material, with a low-melting temperature constituent: they have mainly been used in the past with applications in rapid tooling. By contrast, it could be stated that full melting of metal powders is now suitable for the production of end-usable metal parts [6,7,8,9,10,11,12,13]. However, the performances of the part, in terms of mechanical properties, residual porosity, dimensional accuracy and surface roughness, is closely related to the complex mechanisms involved in heat adsorption and transmission of powders and in melting and consolidation of powders [14,15,16,17].

Various European companies produce machines based on laser systems for direct melting or sintering of metal powders beds [18]. In this study, a direct metal laser sintering (DMLS) machine from EOS GmbH able to process reactive materials, such as cobalt–chromium, titanium and even aluminum alloys, thanks to its laser power and to the inert atmosphere in the building chamber, has been used. Aluminum powder, in particular, broadens the range of possible applications of DMLS to lightweight structural components. To be confident about designing parts for structural applications with this technological process and the material selected, using, for example, FEA (Finite Element Analysis), it is fundamental to have a mechanical characterization of the parts that can be fabricated in different orientations with respect to the powder deposition plane. Very recently, Brandl *et al.* investigated the microstructure of samples manufactured by a Selective Laser Melting (SLM) using an AlSi10Mg powder alloy with a Trumpf TrumaForm LF130 machine: these samples were fabricated for high cycle fatigue and machined afterwards [19]. Buchbinder *et al.* [20] explored the use of newly designed SLM machine equipped with a high power laser up to 1 KW, focusing on the increase of building rate performances, thus, on the laser parameters settings. Olakanmi *et al.* [21,22] investigated the effects of particle size distribution, particle packing arrangement and chemical constitution on the laser sintering of hypoeutectic Al–Si powders and the effect of the processing parameters on the densification mechanism and microstructural evolution in laser sintered Al-12Si powders. Another research group explored the feasibility of introducing high strength aluminum alloys for industrial applications, concentrating mainly on the production of custom powder systems with different particle sizes and different distributions of elementary components [23]. In addition, common materials science literature has some references to the use of aluminum and its alloys for metal matrix composites by additive manufacturing, even though this route is still at an initial stage [24,25,26]. On the basis of the previous considerations, the present work deals with an experimental characterization of an AlSiMg alloy starting from the commercial powders distribution and chemical analyses through to the estimation of the mechanical properties of parts produced with a DMLS machine in four different building orientations and, subsequently, post-treated only by means of shot-peening. Hence the effect of the DMLS process on the microstructure of the final components before and after tensile tests was evaluated by light and electron microscopy.

## 2. Materials and Methods 

The AlSiMg powders supplied by EOS Gmbh were characterized by a Field Emission Scanning Electron Microscope (FESEM, Zeiss SupraTM 40) in order to evaluate their shape and dimensions and then by means of laser granulometry (Fritsch model Analysette 22 Compact) to estimate their size distribution (with volume assumption). The powder chemical composition was assessed through an Inductively Coupled Plasma (ICP) test, in compliance with ISO/IEC 17025; this type of analysis reveals the percentage in weight of the main alloying elements. 

**Figure 1:**
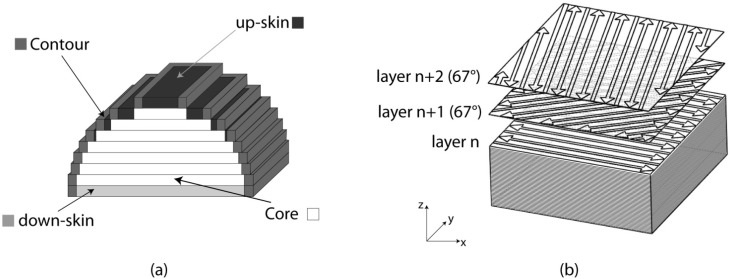
The scanning strategy employed in this work: (**a**) the parameters considered with different power and scanning speed; (**b**) the non-contour area of each layer is rotated of 67° in comparison to the previous one.

The aluminum alloy specimens for the physical and mechanical characterization were prepared by DMLS with an EOSINT M270 Xtended version. In this machine, a powerful Yb (Ytterbium) fiber laser system in an Ar atmosphere is used to melt powders with a continuous power up to 200 W. The detail of the DMLS process, together with the choice of the process parameters to obtain a part with the highest density and the best surface finishing, were described in an earlier study [27], and the values are given in Table 1. As explained in this previous study, the machine employs different parameters for the core of a part, for its lower and upper surfaces parallel to the building plane and for the lateral outer surface, called the contour, as illustrated in Figure 1a. The core and the skin correspond to 2-dimensional surfaces scanned by the laser source, while the contour corresponds to a 1-dimensional closed-type line. First of all, the contour of the layer structure is exposed; then, all of the inner area delimited by the contour is scanned through hatching: the laser beam moves line after line several times (Figure 1b), and the distance between the lines is called the hatching distance. Finally a second exposure of the exterior part contour is carried out to make sure that the part edges correspond exactly to the CAD data, and that part can thus be built with the correct dimensions.

Layer thickness and scanning strategy are also fundamental parameters. The thinner the powder layer, the greater the degree of interlayer bonding and, so, the higher the final density that can be obtained. However, if a too small value is chosen, the speed of manufacturing (and, therefore, the cost) become too slow. 

As regards the scanning strategy associated to the core and to the skin, a certain degree of rotation between the layers leads to a better overlapping of these. This should make the properties of the parts obtained more isotropic in comparison with more conventional scanning strategies made of layers with unidirectional vectors or at least with a cross-ply pattern. As shown in Figure 1b, in this study, the direction of scanning is rotated of 67° between consecutive layers. The skin is made up of three layers.

**Table 1:** Direct metal laser sintering (DMLS) process parameters employed.

Parameters	Skin	Core	Contour
Scan speed (*v*) [mm/s]	900	800	900
Laser power (*P*) [W]	120	195	80
Hatching distance (*h*_d_) [mm]	0.1	0.17	–
Layer thickness [μm]	30	30	–
Laser spot size [mm]	0.01	0.01	0.01

**Figure 2:**
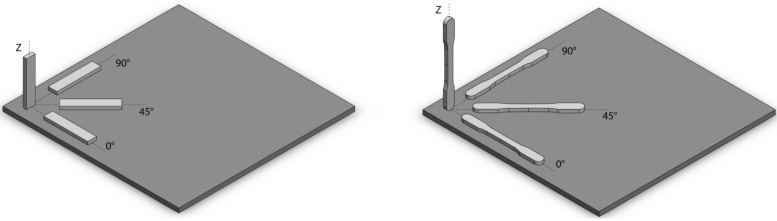
The four different orientations considered for producing DMLS aluminum alloy specimens.

All samples for physical and mechanical evaluations were fabricated along different orientations using the above mentioned optimized parameters and scanning strategy. The orientations considered (see Figure 2) are along the *z* axis, called the “build direction”, and along three directions on the building platform (*xy*-plane): parallel to the direction of the powder deposition (0°), along the normal axis to it (90°) and at 45° between them. Samples of rectangular shape and 50 × 10 × 5 mm size were produced to analyze the density, hardness and Young’s Modulus (Figure 2a). Considering tensile tests, five specimens for each orientation were built according to the standard ASTM E8M and along the above described orientations (Figure 2b). To verify the reliability and reproducibility of the process, the fabrication of these samples was repeated twice. 

Due to the high thermal gradients, this process fabricates parts with high residual thermal stresses. In order to avoid the bending of the parts, before removing them from the building platform, it was necessary to perform an annealing for 2 h at 300 °C. The samples were then shot-peened (Ecoblast/F machine, by Silco S.r.l.—Italy) to improve the surface quality and integrity. As a result of previous analysis [27], in this study, glass beads of 200 µm diameter with an air pressure value of 8 bar for several seconds were used. These beads produce a clean, bright, satin-finished surface, with an *R_a_* average value of 3 μm without dimensional change or contamination of the parts. 

The density of the specimens was measured by the water displacement method (Archimedes), and the measurements were expressed using the mean value. The standard deviation is less than 0.05 g/cm^3^. Then, samples were polished down to 1 μm diamond paste to allow Vickers microhardness measurements performed by a Leitz instrument (load 50 g for 30 s): fifteen measurements were done to calculate a mean hardness value for each orientation. Elastic modulus was evaluated by an impulse excitation technique involving the analysis of the transient natural vibration, by means of a GrindoSonic MK5 instrument, according to the standard ASTM C1259. For microstructure analyses, the DMLS samples were cross-sectioned perpendicular and parallel to the building platform, then polished down to colloidal silica suspension (size 0.05 μm) and etched with Weck’s reagent (KMnO_4_ and NaOH in distilled water) for 15 s; after that, they were observed by an optical microscope (Reichert Young MF3) and by FESEM. All the microstructural images refer to the core of the parts. 

Finally, tensile tests were performed on an EASYDUR 3MZ—5000 testing machine, with a free-running crosshead speed of 2 mm/min: strain was measured by a piezo-electric extensometer. After rupture, the fracture surfaces were observed by Field emission scanning electron microscopy (FESEM). 

## 3. Results

### 3.1. Powder Characterization

**Figure 3:**
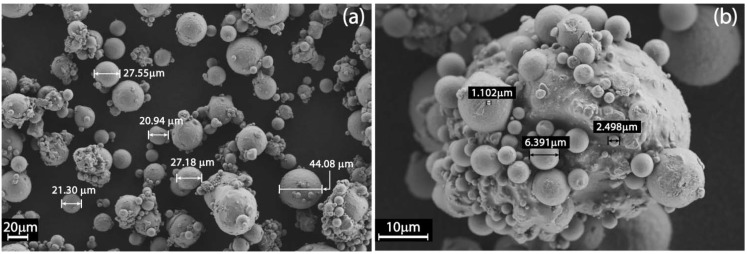
The as-received aluminum alloy powder observed by field emission scanning electron microscope (FESEM).

As illustrated by Figure 3a, the as-received gas atomized aluminum alloy powder particles are spherical and quite regular in shape, ranging in dimensions from 1 to 44 μm, with an average around 21–27 μm. 

The numerous smallest particles, with mean diameters less than 10 μm, tend to agglomerate on the surface of the bigger ones (Figure 3b), creating some clusters of about 60 to 80 μm, and this could be detrimental for the final density of the DMLS parts, considering that the layer thickness employed in this research is of 30 μm. 

**Figure 4:**
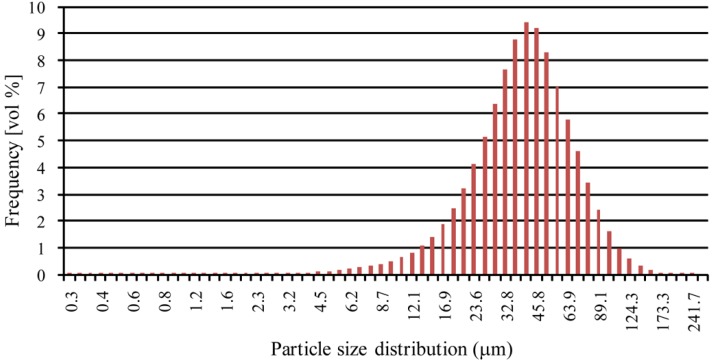
Particle size distribution of as-received aluminum alloy powder.

This tendency is confirmed by the granulometric analyses reported in Figure 4. The diameters corresponding to 10% (d_10_), 50% (d_50_) and 90% (d_90_) of the cumulative size distribution are 19.3 μm, 40.7 μm and 74.8 μm, respectively. In the graph of Figure 4, the frequency distribution is based on a volumetric assumption. This means that even if the small particles are far more than the bigger ones in number, their mean volume is three orders of magnitude less than the big ones: for this reason, they could not be displayed. 

In addition to the particles size and distribution, also their chemical composition could affect the densification in DMLS process, as observed by Olakanmi *et al.* [22]. The values of the chemical analyses conducted via ICP test are summarized in Table 2. As can be seen, the main alloying elements are silicon and magnesium, as expected, but there is also an appreciable quantity of iron. 

**Table 2:** Chemical composition of aluminum alloy powder as determined by inductively coupled plasma (ICP) test.

Element	Weight (%)
Si	10.08
Fe	0.16
Cu	0.001
Mn	0.002
Mg	0.35
Zn	0.002
Ti	0.01
Al	balance

### 3.2. Mechanical Characterization

In Table 3 are summarized the mean values for density, Young’s modulus and Vickers Hardness. Moreover, considering for the AlSiMg alloy a theoretical density of 2.68 g/cm^3^ [28], the percentage of the residual porosity can be calculated: it ranges from 0.7% to 0.8%, so it is indeed low. 

**Table 3:** Density, Hardness and Young’s Modulus of aluminum alloy DMLS specimens.

Orientation	Density (g/cm^3^)	Residual Porosity (%)	Hardness (HV)	Young’s Modulus E (GPa)
*xy*-plane	2.66	0.8	105 ± 2	73 ± 1
*z* axis	2.66	0.7	108 ± 3	72 ± 1

It was observed that the AlSiMg DMLS samples have isotropic properties when built on the *xy*-plane, as expected from the scanning strategy adopted in this study. For this reason, a single mean value for each property is reported in the table. Considering the samples built along the *z* axis direction, there are some differences, but they could be considered negligible.

Hardness is well recognized as a first indication for mechanical properties: using micro-Vickers, the measurements could be done relatively close to each other, making it possible to investigate the change of hardness of the DMLS parts with depths. The mean value profiles obtained along the total length of four rectangular bars for each orientation are very close and with flat trends. Thus, in a first approximation, it can be assumed that the samples were also homogeneous in terms of microstructure along the whole section. 

Considering tensile tests, the results are summarized in Table 4. Variations were not found among performances of samples with different orientations on the powder deposition plane, while there are some differences with the values obtained along the direction perpendicular to it.

**Figure 5:**
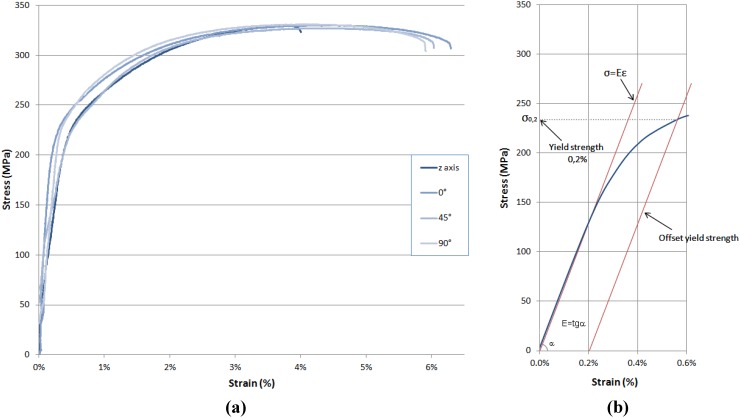
(**a**) Typical stress-strain curves for aluminum alloy DMLS specimens built along four different orientations; (**b**) yield strength and Young’s modulus evaluation for the representative curve of a specimen along *z* axis.

In Figure 5a are shown the representative trends of stress-strain curves for each orientation considered. As can be seen, the results are well reproducible. In Figure 5b, it is graphically explained how the yield strength values were calculated: this is shown for a typical stress-strain curve of a sample along the build direction. Considering the tangent to the curve in the elastic region, the values for the Young’s modulus could also be estimated: it was confirmed that they are in good agreement with the results obtained by the impulse excitation technique.

**Table 4:** Mean values of tensile properties of aluminum alloy DMLS specimens produced according the standard ASTM E8M along different orientations, compared to a similar alloy as-fabricated.

Material	Orientation	Yield Strength σ_0.2_ (MPa)	Ultimate Tensile Strength σ_UTS_ (MPa)	Elongation at break (%)
**AlSiMg after DMLS**	*xy*-plane	243 ± 7	330 ± 3	6.2 ± 0.3
	*z* axis	231 ± 3	329 ± 2	4.1 ± 0.2
A360.0 F ^*^	–	170	317	5

* Temper and product form: as-fabricated [28].

### 3.3. Microstructure and Fracture Surface Analyses

**Figure 6:**
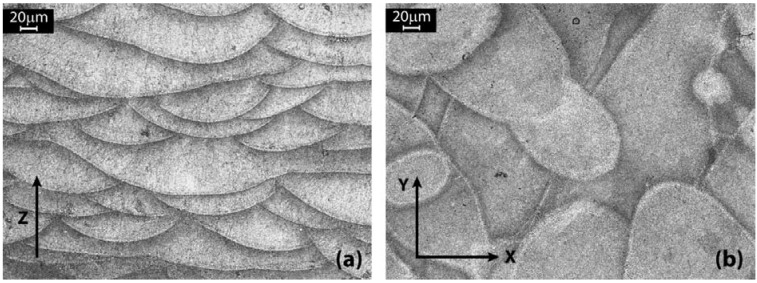
Optical microscope images of an aluminum alloy DMLS sample after etching with Weck’s reagent: (**a**) a section along the build direction (*z* axis); (**b**) a section parallel to the powder deposition plane (*xy*-plane).

The AlSiMg DMLS samples microstructure was initially analyzed by light microscopy. Figure 6 shows micrographs of two cross sections: a section parallel to the build direction, indicated by the black arrow (Figure 6a), and a section perpendicular to it (Figure 6b). The images refer to the core of the parts.

The etching with Weck’s reagent is useful to highlight the molten pools and their contours. During the process, upon irradiation, the powder material is heated, melts and forms a liquid pool. Afterwards, the molten pool solidifies and cools down quickly, and the consolidated material starts to form the product. After a complete layer is scanned, the building platform is lowered by an amount equal to the layer thickness, and a new layer of powder is deposited, following a pattern rotated 67° around *z* axis, as illustrated in Figure 1. 

The laser beam intensity is modulated in such a way as to ensure that the new powder layer is melted and penetrates the previous layer, so remelting it to accomplish a good connection of the layers (wetting of the layer underneath) at the same time. Therefore, considering a section along the build direction, these melt pools are all oriented in the same way (Figure 6a), being a section made by the superimposition of subsequent layers. However, due to the scanning strategy adopted in this study and in account of the above mentioned partial re-melting, the shape of these melt pools is not simply half-cylindrical. Consequently, in this case, it is not possible to define their mean depth. On the other hand, considering the section parallel to the powder deposition plane, it could be assumed to observe the cross-section of melt pools of different layers (Figure 6b): due to the different depth, their contours overlap originating shapes with irregular geometries. 

**Figure 7:**
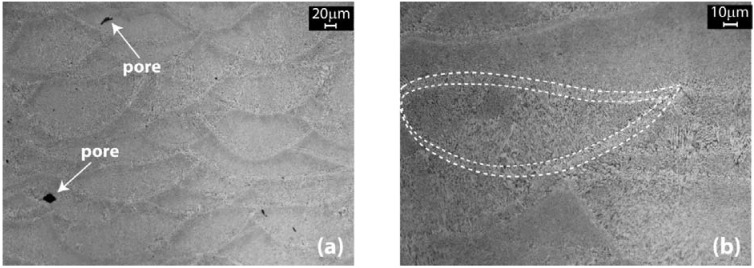
Optical microscope images after Weck’s etching showing: (**a**) the presence of pores and at a higher magnification; (**b**) the shape of a melt pool with its contour**.**

Looking at the micrographs of Figure 6, even if some porosity is visible in the samples investigated, the pores dimension is very little. Going at higher magnifications, it is possible to appreciate their mean size: in Figure 7a, two pores are visible, with a dimension of about 20 μm, while Figure 7b is focused on a single melt pool and its region of contour. As can be observed, the rapid and localized melting and cooling of DMLS originate very fine microstructures. 

**Figure 8:**
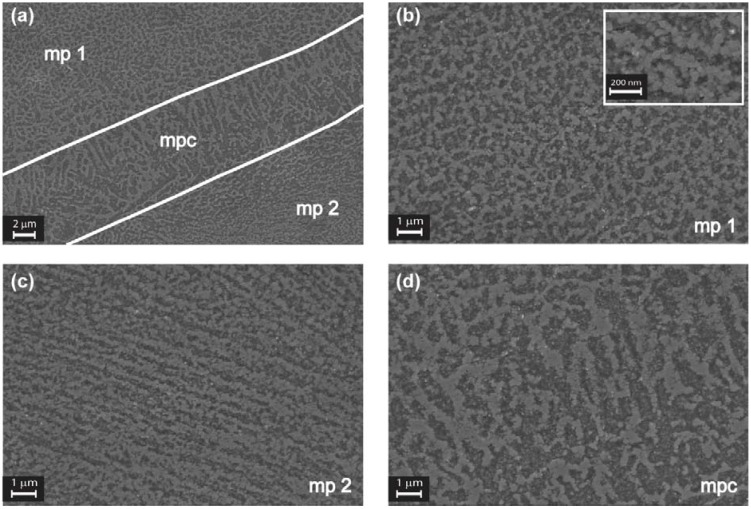
FESEM images of an aluminum alloy DMLS sample after etching with Weck’s reagent, sectioned along the *z* axis: (**a**) mp_1_ and mp_2_ are areas of two adjacent melt pools, while the region delimited by the white lines correspond to the melt pool contour (mpc); (**b**–**d**) the three regions at higher magnifications. In the inset, it possible to observe the nanometric grain size.

To appreciate this small grain size, the section along the build direction was observed by FESEM, focusing on a region between two melt pools, as shown in Figure 8a. The area inside the white parallel lines corresponds to the melt pool contour (mpc), with a mean height of about 8 μm. At higher magnifications, from Figure 8b–d, it can be seen that the three regions (mp_1_, mp_2_ and mpc) are characterized by a fine cellular-dendritic structure made by agglomerates of grains with mean diameters of 80 nm or less. It was found that these agglomerates are different in length, thickness and aspect ratio in the three regions. 

According to Olakanmi *et al.* [21] it can be assumed that there is little or no free-energy barrier to the phase transformation from liquid to solid. It is due to the complete wetting of the substrate by the molten metal and the nearly ideal interface provided by the partially melted heat affected zone (HAZ) grains at the fusion boundary. In this way, the grains grow epitaxially, and the grain direction is parallel with the local conductive heat transfer, as shown in Figure 8b–d. 

**Figure 9:**
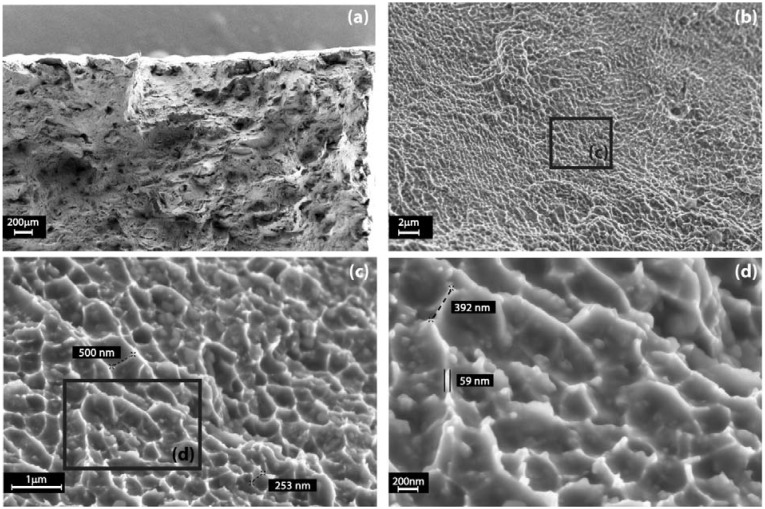
FESEM images of an aluminum alloy DMLS fracture surface at increasing magnifications: (**a**–**d**) it is covered by sub-micrometric voids and dimples with a nanometric thickness.

Also, fracture surfaces after tensile tests were investigated by FESEM, as illustrated by Figure 9. It may be assumed that all the samples failed, because of ductile fracture as the result of growth and coalescence of micro-voids. In fact, as observed at higher magnifications reported in Figure 9c,d, the fracture surface is completely covered by very fine dimples, demonstrating also a great ability to dissipate the energy of fracture. It is possible to appreciate the micro-voids dimensions, from 250 to 500 nm per side, and the dimple thickness, of about 60 nm, a peculiarity of this process.

## 4. Discussion 

From the combination of FESEM and granulometric analyses, it could be stated that the AlSiMg powders employed in this study are made of a large amount of very small spheres and only a few bigger ones. As observed by Simchi [9], finer particles provide a larger surface area to absorb more laser energy, leading to a higher sintering rate. However, before starting the additive process, it is fundamental to sieve the powder to ensure a maximum particle size in agreement with the layer thickness when filling the DMLS machine. Considering the powder chemical composition and comparing it to the literature [28], it could be assumed that this aluminum alloy is similar to the A360.0 alloy, in particular A360.2, which has a nominal density of 2.68 g/cm^3^ and a melting temperature around 570 °C, due to its near-eutectic composition. This alloy is a typical casting alloy used for example for parts with thin walls and complex geometry. Even if the as-received powders are gas atomized under a nitrogen atmosphere, a thermal treatment for drying them at 80 °C for five hours was conducted prior to perform the DMLS process in order to avoid any traces of humidity.

Regarding the mechanical properties investigated (Table 3 and Table 4), it could be assumed that they are isotropic for samples built on the building platform plane and slightly different for samples built along a direction perpendicular to it. In particular, if compared with the commercial A360.0 alloy in as-fabricated conditions [28], AlSiMg DMLS specimens show very high values of yield strength, with an enhancement of about 43% for samples built in the *xy*-plane and 36% for samples along the *z* axis. The ultimate tensile strength is a little higher, while for the elongation at break, there is an enhancement on the *xy*-plane and a decrease along the build direction, as already observed in literature by Tolosa *et al.* [29]. It is well known that this is the weakest direction for samples produced by DMLS. However, the elongation at break is only slightly lower than conventionally processed material. 

During the DMLS process, on account of the short interaction times and high conductive heat transfer rate, a very fine microstructure originates. The exposure period of the laser irradiation is in the range of milliseconds. In fact, the process can be considered as “high power density-short interaction time” [9]. The very high cooling rates, ranging between 10^3^ and 10^11^ K/s [30], promote greater under-cooling, thus producing finer grains and a gradual change in the solidification regime from dendritic to cellular-dendritic. At the same time it is difficult to ascertain the nature of the small amount of precipitates that could be formed due to the non-equilibrium conditions and the solidification conditions associated with the DMLS process. In this study, using the AlSiMg alloy described, Si and Mg_2_Si precipitates were expected, but probably they were removed preferentially by the chemical etching employed.

However, looking also at the fracture surfaces analysis, it can be concluded that this very fine microstructure is responsible for the superior overall mechanical properties without the need of a further heat treatment, as usually happens with casting alloys.

## 5. Conclusions 

After characterization and analysis of the starting powders’ size, morphology and chemical composition through to the evaluation of mechanical properties of DMLS as-built specimens, along with microstructural observations, it can be concluded that:

- The AlSiMg powders employed in this study are spherical in shape with an average size of 21–27 µm, but very fine particles with a diameter lower than 10 µm tend to agglomerate, forming bigger clusters of irregular shape. These clusters can adversely affect the flowability of the powders: so, it is fundamental to sieve them before starting the DMLS process.
- Density evaluation of samples indicates a residual porosity of about 0.8%. Microscopic observations show that porosities are very small, on the order of 20 to 30 µm.
- In comparison to the properties of a commercial as-cast A360.0 alloy, AlSiMg DMLS specimens show very high values of yield strength, with an enhancement of about 43% for samples built in the *xy*-plane and 36% for samples along the *z* axis. The ultimate tensile strength is slightly higher in both cases, while for the elongation at break, there is an enhancement on the *xy*-plane and a small decrease along the build direction.
- Microstructure observations show a very fine cellular-dendritic structure by agglomerates of grains with mean diameters of about 80 nm, responsible for the superior overall mechanical properties without the need for a further heat treatment.
- Fracture’s surfaces analysis reveals also a very peculiar behavior: the fracture was caused by coalescence of submicrometric voids, with dimples of nanometric thickness.

This analysis could be the base to properly design lightweight structural components in AlSiMg through DMLS by means of a FEA, for envisaged applications in different fields, such as aerospace, automotive and robotics.
